# 

**Published:** 1884-01

**Authors:** 


### CONTENTS.

Biographical........................................................................ 7

COMMUNICATIONS.

Prosthetic Dentistry........................................................................ 13
On Erosion........................................................................ 18

SELECTIONS.

The Value of Definite Therapeutical Combination........................ 37
Removal of the Greater Portion of Both Upper Jaw-Bones without External Incision........................................................................ 39
The Lack of Interest in Hygiene........................................................................ 40
Miscellany........................................................................ 40
A Protest Against Changes in Nomenclature........................................ 42
Sugar as a Dressing for Wounds........................................................ 42
The Common Diseases of Children........................................................ 44
Correspondence........................................................................ 45
Salmagundi........................................................................ 47

EDITORIAL.

A Good Antiseptic........................................................................ 52
Vick's Floral Guide........................................................................ 53
Suspended........................................................................ 55
A New Departure........................................................................ 55
Physicians' Visiting List for 1884........................................................ 56
Personal........................................................................ 56
A New Clamp........................................................................ 56
Resolutions on the Death of Dr. A. L. Talbert........................................ 57

